# Do dietary habits and iron-folic acid intake make a difference? Factors associated with anemia in pregnancy: a multi-center cross-sectional study

**DOI:** 10.3389/fgwh.2025.1599842

**Published:** 2025-06-30

**Authors:** Serawit Lakew Chillo, Endrias Markos Woldesemayat, Mesay Hailu Dangisso

**Affiliations:** ^1^ School of Public Health, College of Medicine and Health Sciences, Hawassa University, Hawassa, Sidama, Ethiopia; ^2^ Ethiopian Public Health Institute, Addis Ababa, Ethiopia

**Keywords:** risk factors, anemia, pregnancy, iron folic acid, south Ethiopia

## Abstract

**Background:**

Anemia in pregnancy impacts the well-being of the women and the conception. Anemia is associated with increased risks of maternal mortality. In Ethiopia, three in ten pregnant women were affected by either mild, moderate, or severe anemia. The recent evidence was limited in the study locations of southern Ethiopia and needs to be updated. No report of anemia as relation to dietary factors observed in the region. This study therefore aimed to assess factors associated with anemia in pregnancy in the region.

**Methods:**

A multicenter, cross-sectional study was conducted from January 2 to 30, 2025, in five selected public health facilities in Arba Minch district, South Ethiopia.

**Results:**

A total of 476 (96%) respondents had participated in the survey. Explanatory variables were modeled in logistic regression to test for statistical associations at a *P* value of <0.05. The study participants were in the age range of 18 to 40 years. Of the respondents who completed the survey, 103 (21.6%, 95%CI 18.2–25.6%) were diagnosed as anemic. Participants who received iron-folic acid during the current pregnancy were 66% less likely to be anemic, AOR (95% CI), 0.34 (0.19, 0.61). Participants with high education status, sleeping under insecticide-treated bed nets (ITN), not being infected with malaria in the current pregnancy, and age category between 20–29 years were less likely to experience anemia in pregnancy. Evidence was limited to support association of women dietary diversity score (WDDS) and anemia in pregnancy, AOR (95%CI), 0.83 (0.49, 1.40).

**Conclusions and recommendations:**

Anemia prevalence was a moderate public health problem in the study area. Healthcare workers should encourage antenatal women to receive iron and sleep under insecticide-treated bed nets (ITN) for anemia protection during pregnancy.

## Introduction

Anemia in women is an extensive public health problem with a huge impact on women's health, economy, and social status (1). In hundred forty countries, anemia in women aged 15 to 49 years remains a moderate to severe public health problems (1). The World Bank Group reported in 2019 that the prevalence of anemia in pregnant women was 39.8% in Eastern and Southern Africa, 45.8% in Sub-Saharan Africa, and 36.5% worldwide (2). Women in developing countries were more affected by anemia during pregnancy than those in the developed world, as related to variations in health, economic, and social factors (3).

The progress toward WHO's 50% global anemia reduction plan of 2025 among women of reproductive age (pregnant and non-pregnant) was not on track (1, 4). The World Bank Group addressed that attaining the global target of anemia reduction needs to scale up micronutrient interventions, delivery platforms, and strong political determinations (1). Due to the inability to achieve the global anemia target, WHO and UNICEF jointly extended the target to 2030 (4). The global target to reach by 2030 is 15.2% anemia prevalence in women of reproductive age (1, 4).

Anemia prevalence among regions varies in Ethiopia. Overall, the national prevalence of anemia in pregnancy was 29% in 2019 (2). A meta-analysis report showed that the lowest prevalence was in Amhara region (15.9%) and the highest in Ethiopia Somali (56.8%) (5). A recent 2023 study report by the Ethiopian Public Health Institute (EPHI) showed 13% of anemia prevalence among women of reproductive age (6), close to a 50% reduction from the 2019 World Bank Group report of Ethiopia's data (7). Across regions of Ethiopia, women in reproductive age anemia prevalence ranges between 6% in Addis Ababa to 40% in Somalia regional state of Ethiopia (6). In the southern nations' nationalities and people state of Ethiopia, the reported prevalence of anemia in women of reproductive age was 10% (6).

The women dietary diversity score (W-DDS) is defined as a dichotomous indicator used to measure foods consumed by a woman (15–49 years) in the last day and night (8, 9). It is considered adequate food diversity when five out of ten food domains are consumed by a pregnant woman in the last day and night (8, 9). The dietary bioavailability of micronutrients (such as, iron) consumed by the women depends on the availability of enhancers (ascorbic acid and animal tissues), inhibitors (calcium, oxalic acid, phytic acid, and polyphenols), and the type of iron in the food (heme/nom-heme) (10). Heme iron is better and absorbed easily than non-heme iron (11). Non-heme iron is mostly found in plant sources; however, heme iron is mostly found in flesh foods (such as red meat) (11). Nearly half of the iron that is found in flesh foods are heme iron (11, 12). Nearly 15 to 35% of heme iron is absorbed by human body and which depends on body needs (13). Around 2%–20% of nonheme iron is absorbed by human body from plant or animal sources (14).

Studies associated anemia in pregnancy with advanced age (15), low education status, being in the second or third trimester of pregnancy, caffeine intake in pregnancy, high family size (15–18), being poor, low dietary diversity score, high gravidity, iron supplementations, and maternal infection with soil-transmitted helminthiasis during pregnancy (17–19). On the other side, a contrasting result was reported that women's higher education status was related to anemia in pregnancy (18). A systematic review report showed malaria infection in pregnancy was associated with anemia in pregnancy (5). Consuming less than three meals per day was another determinant of anemia in pregnancy (18). There was no recent evidence that shows factors associated with anemia among pregnant women in the Southern Ethiopia Regional State. This study therefore assessed socio-demographic, food, and behavioral factors associated with anemia during pregnancy.

## Methods and materials

### Study location and design

This study was performed in Arba Minch district, Southern Region of Ethiopia (20). The district had one referral and teaching hospital, one general hospital, one primary hospital, and 7 primary health care units (health centers).^1^ The study area is located in the Great Rift Valley, at a 450-kilometers road distance from the capital of Ethiopia, Addis Ababa. The altitude of the study location ranges between 1,090 to 1,200 meters above sea level (21) and the annual temperature ranges between 16 and 37°c (22). Based on projection data for 2022, the population of the nearby rural study district was 214,020 with a population density of 221.2/km^2^ and the study town population was 201,049 with a density of 6,098/km^2^ (23). The estimated population of women in the reproductive age group in the district was 96,711, and the estimated number of annual pregnancies was 14,361 (21, 23). The design of this study is an institution-based, multicenter, cross-sectional survey undertaken from January 2 to 30, 2025.

### Sampling estimation and procedure

**Figure 1:**
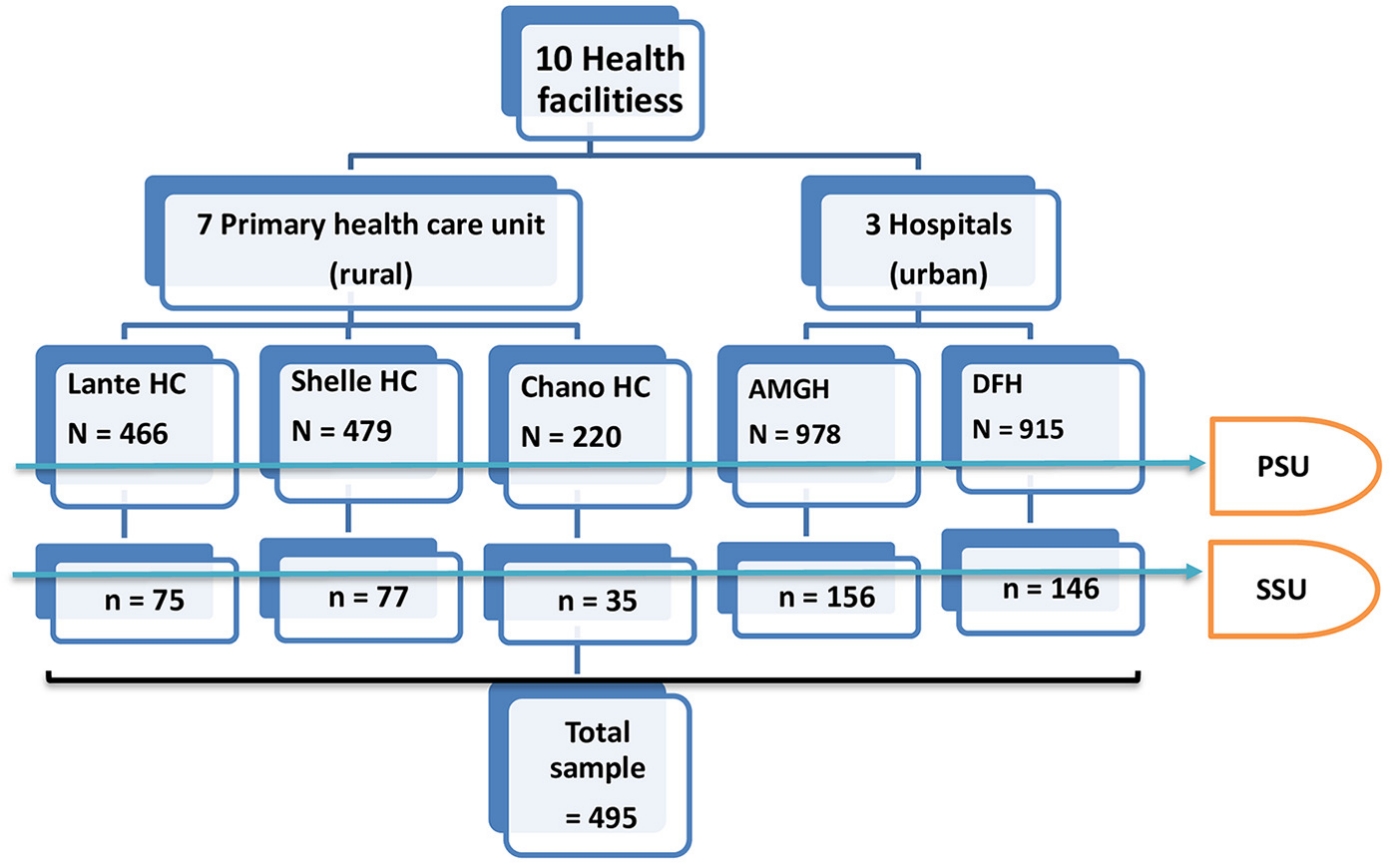
The participant recruitment and selection flowchart. *N*, total population of antenatal women in a month; *n*, sample population; HC, health center; AMGH, arbaminch general hospital; DFH, dil fana primary hospital; PSU, primary sampling unit; SSU, secondary sampling unit.

To determine minimum sample size, the single population proportion formula was employed. The assumptions used in the sampling estimation process were a 95% CI, a design effect (1.5), a non-response rate (5%), and the prevalence of anemia among ANC attendants at Dilla University referral hospital, South East Ethiopia, was 28.7% (24). The minimum number of respondents required to be interviewed was 495. The study was conducted in five selected public health facilities among ten. A stratified two-stage sampling technique was employed, where health facilities were primary sampling unit and respondents in the selected facilities were secondary sampling unit (25). Facilities were stratified as urban and rural setting, such as two hospitals selected from urban and three health centers from rural setting. Respondents were proportionally assigned to each of the five selected health institutions as per their monthly average population of pregnant women who visit for antenatal services. Systematic sampling was employed to achieve the desired sample size in each of selected facilities. The variance across the urban/rural strata was assumed equal (26). This was because pregnant women in either of the urban or rural health facility could make antenatal visit from either urban or rural dwellings. The average number of pregnant women per month was obtained from selected facilities' monthly antenatal reports to calculate the proportion. The selected health care settings were Arba Minch Hospital (AMGH), Arba Minch Dil Fana Hospital (DFH), Chano Health Center, Kola Shelle Health Center, and Lante Health Center (Figure 1).

### The study population and eligibility criteria

The study population were antenatal women who were under ANC follow up in the public health facilities of Arbaminch district and town during the study period. The target population were pregnant women selected and participated in this particular study during the study period. During the enumeration process the eligible women were any pregnant women who were willing to participate, exist in any of the frequency of antenatal visit, permanent resident (lived a minimum of 6 months in the setting), and no any mental and physical illness during data collection.

### The data collection process and measurement

The data collection tool was structured, validated, and pre-tested before the start of the actual data collection process. The questionnaire was adapted from the Ethiopian DHS 2016 (27) and literature sources (19, 28). Interviewers interviewed respondents who had visited each study facility consecutively until the sample size was achieved. Data was collected based on two approaches. First, participants were interviewed face-to-face for interview questions. Next, the participant's laboratory result was used to extract data on the anemia status of women. The KoboToolbox was used to collect data electronically using mobile tablets (29). The client's medical record was used to collect anemia data. The enumerator administered the respondent's response and data from the health facility card. Two experienced supervisors who had a BSc degree in nursing were hired. Eight experienced data collectors who had a BSc degree in nursing were hired. The total data collection period was one month. The explanatory variables include social, demographic, economic, reproductive, and other variables [such as IFA and deworming that the participant received in the current pregnancy, history of malaria and intestinal parasitic infections in the current pregnancy, health insurance benefit status, sleeping status under ITN in the current pregnancy, and BMI (KG/M^2^)]. Socioeconomic and demographic variables include the participant's age, marital status, education and literacy level, occupation, urban-rural residence status, smoking status, and household income status. Reproductive characteristics were parity, gravidity, menstrual disorders, inter-pregnancy intervals, and mode of delivery. The dependent variable was the anemia status of women, which was measured as anemia [HGB < 11.0 g/dl at sea level (2)]. An altitude adjustment of −0.2 g/dl was made to the HGB level of participants based on UNHCR guidelines (30).

#### Sample collection and procedure to detect HGB level

Laboratory technicians collected a 3 ml whole blood from a participant venous blood of the upper left hand. The aim of blood sampling was to do laboratory analysis to detect blood HGB level (anemia status) using a CBC machine (mindray-2017, BC-3000Plus). For the blood sample collection, the anticoagulant K3 ethylenediamine-tetraacetic acid (k 3 EDTA) tube was used. There was no sampling storage in the process as the collected sample was analysed immediately (within 2 h) in the study health facilities. The CBC machine used for laboratory analysis was precalibrated by biotechnologists in the study facility. Sample collectors and laboratory investigators were recruited as medical laboratory technicians in the MCH clinics.

### Women's dietary diversity score

The respondents were told to recall the food they had received the last day and night before the day of the survey. Even-though this 24-hour recall was limited to capture a short-term intake rather than a habitual intake, the recall bias was minimized. The list of foods was categorized under 10 FAO (9) dietary categories, such as grains, white roots and tubers, and plantains (category1); pulses (beans, peas or lentils) (category2); nuts and seeds (category3); dairy (category4); flesh foods (category5); eggs (category6); dark green leafy vegetables (category7); vitamin A-rich fruits and vegetables (category8); other fruits (category9); and other vegetables (category10). The detailed list of foods under each category was mentioned in the questionnaire. If the respondent consumed foods in the list with the category that the interviewer reads, “yes” was reported; otherwise, “no” was reported. Any of the “yes” responses in the list of each dietary category was considered as “yes” to the category. When the “yes” response in each of the 10 dietary categories was 5, it was considered an adequate minimum dietary diversity score (MDD-W) for women (9). Whereas the score of 0–4 was considered as an inadequate MDD-W score for women (9).

### Data quality control

Before the actual data collection, the enumerators and supervisors were given three days of training. Training included data collection tools word by word, study methods, and the KOBO toolbox application utilization in the tablet. A mock interview was done between data collectors. A pretest was given to 25 antenatal women in the health centers not selected for this study, such as Arba Minch Secha, Limat, and Zigiti Bakole. Every day, field supervisors checked for completeness, missings, and challenges faced during the interview. The supervisors had given feedback to data collectors before the initiation of next-day data collection. The local language (Amharic and Gamogna) was used in the communication during the data collection process. The local language was back-translated to the original language (English).

### Analysis

The collected respondent data was accessed from the KOBO server and imported to Stata 17 statistical software package for statistical analysis. Summarized results are reported in figures and tables. The participants' characteristics and categories were compared between anemic and non-anemic respondents in the tables and figures. The unadjusted logistic regression model was fitted to explanatory variables to check for unadjusted statistical associations. Statistically significant variables by unadjusted (*P* value <0.25) were fitted in to an adjusted logistic regression to control for confounding effects. The final statistical association was declared in the adjusted model at a *P* value <0.05. To show strength of statistical associations, the precision with 95%CI was reported for adjusted and crude odds ratio. The better model was selected based on log likelihood statistics (−214.93). The better model was selected as per the higher log likelihood.

## Results

### Basic characteristics

A total of 476 (96%) respondents had completed the survey. The mean age (years) and standard deviation (SD) of the participants was 26 ± 5.0, where it ranges between 18 and 40 years. A total of 457 (96%) respondents were married and lived with their husbands. Nearly one-third of participants [148 (31%)] had completed college. About two-thirds [338 (71%)] of participants were permanent urban residents. The median daily household income of respondents was $1.59 US, with a maximum of $19.84 US and a minimum of $0.08 US. Nearly two-thirds, 305 (64%), of study participants were not a member of the community-based health insurance scheme (Table 1).

**Table 1:** Socio-demographic characteristics of study participants, Arba Minch district, South Ethiopia, *n* = 476, January 2025.

Variables and category	Frequency	Percent
Age group (years)		
15–19	34	7.1
20–29	298	62.6
>29	144	30.3
Marital status		
Married	457	96.0
Others^a^	19	4.0
Level of education		
Primary or no formal school	176	37.0
High school	152	31.9
More than high school	148	31.1
Occupation		
Farmer	80	16.8
Government employed	76	16.0
Merchant	96	20.2
House wife	164	34.5
Others^b^	60	12.6
Place of residence		
Urban	338	71.0
Rural	138	29.0
Daily household income in $US		
<1.25	140	29.4
1.25–2.5	207	43.5
2.5–4	85	17.9
>4	44	9.2
Household health insurance status		
Insured	171	35.9
Not-insured	305	64.1

^a^ Single and divorced.

^b^ Private employed, house worker, daily laborer, and student.

$US, united states dollar.

### The reproductive and other personal characteristics of respondents

One out of ten respondents, 49 (10.3%), had reported having a history of heavy menses. The majority of respondents reported having no history of intermenstrual bleeding at any time in their lives, 390 (82%). The median number of births a respondent ever had was 1.0, which ranges between a minimum of 0 and a maximum of 6. The median number of pregnancies a respondent ever had was 2.0, which ranges between 1 and 8. The median interpregnancy interval was 4 years, which ranges between a minimum of <1 year and a maximum of 20 years. Close to half of respondents [231 (48.5%)] received IFA supplements in the current pregnancy and consumed tea/coffee within 30 min before or after meals [208 (44%)]. One-thirds of respondents [158 (33%)] were intentionally avoided to eat some useful food types during pregnancy, such as fish, meat, cabbage, roots, maize, egg, and banana. “The reason for avoiding some foods” was added in the manuscript, such as “The reason reported for avoiding some foods” were due to insulting foods with no reason, nausea, vomiting, dislike food odor, gastric discomfort, gastric reflux, food not allowed in pregnancy, and respondent do not know why avoided (Table 2). In the current study locality with high malaria risk category (31), respondents reported malaria infection history any time during the current pregnancy was 62 (13%). However, majority of the respondents reported as slept last night under ITN, 346 (73%) (Table 2).

**Table 2:** Participant's reproductive and other personal characteristics in the study region of south Ethiopia (*n* = 476), January 2025.

Variables	Category	Frequency	Percent
History of heavy menstrual bleeding	Yes	49	10.3
	No	427	89.7
History of intermenstrual bleeding	Yes	86	18.1
	No	390	81.9
Parity	0–1	277	58.2
	2–3	135	28.4
	4+	64	13.4
Gravidity	1	137	28.8
	2–3	225	47.3
	4+	114	23.9
History of stillbirth	Yes	46	9.7
	No	430	90.3
Interpregnancy interval (*n* = 339)	<24 months	16	3.4
	24–36 months	143	30.0
	>36 months	180	37.8
IFA received in the current pregnancy	Yes	231	48.5
	No	245	51.5
Consumed tea or coffee within 30 min before or after a meal	Yes	268	56.3
	No	208	43.7
Intentionally avoiding some useful foods in the current pregnancy	Yes	158	33.2
	No	318	66.8
Reason for avoiding some useful foods (*n* = 158)			
Insulting foods with no reason		55	34.8
Nausea		23	14.6
Vomiting		21	13.3
Dislike food odor		15	9.5
Gastric discomfort		12	7.6
Gastroesophageal reflux		11	7.0
Believes that food not allowed in pregnancy (fears baby overweight)		5	3.2
Do not know why avoided foods?		16	10.1
Meal frequency in the current pregnancy	2 or 3 meal and no snack	192	40.3
	3 meal and 1 snack	202	42.4
	3 meal and 2 or more snack	82	17.2
History of malaria in the current pregnancy	Yes	62	13.0
	No	414	87.0
Slept last night under ITN	Yes	346	72.7
	No	130	27.3

ITN, insecticide treated net; IFA, iron folic acid.

### Dietary habits of study participants

**Figure 2:**
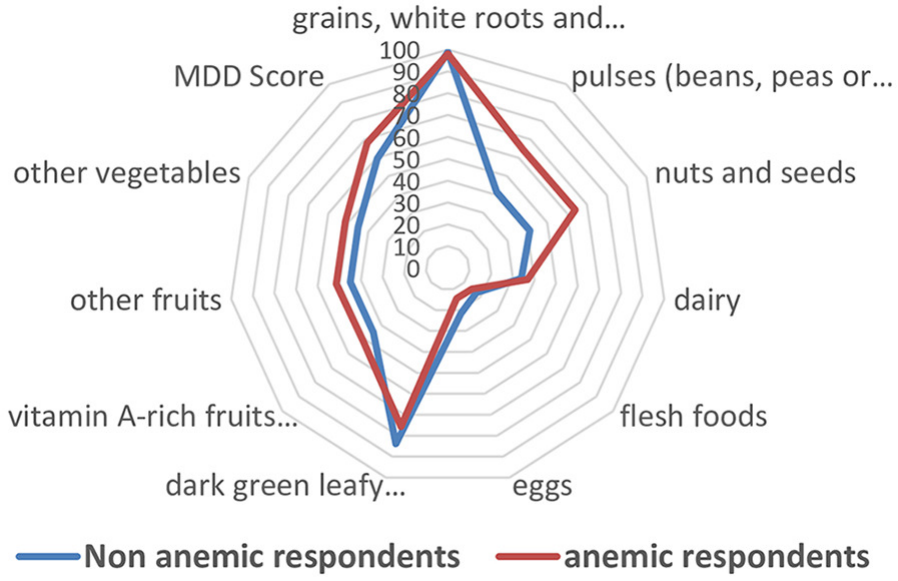
Percentage of the women who consumed foods in the last 24 h before the day of the survey vs. anemia status, Arba Minch district, South Ethiopia, January 2025.

As shown in Figure 2, participants adequacy of minimum dietary diversity score (MDD) was similar between anemic, 70 (68.0%), and non-anemic respondents, 222 (59.5%), *P* value = 0.12. Overall adequate MDD score among the study participants was 292 (61.3%). Nearly all respondents, 470 (98.7%), had consumed grains, white roots and tubers, or plantains the last day and night before the day of the survey. However, few respondents consumed flesh foods (such as organ meat, fish, and meat), 79 (16.6%). The dairy products were consumed by three out of ten participants 165 (34.7%), and eggs consumed by one out of five participants 96 (20%). Many participants consumed dark green leafy vegetables, 391 (82.1%), and nearly half of the respondents consumed vitamin A-rich fruits and vegetables (such as ripe papaya, mango, yellow/orange-fleshed banana, melon, orange-fleshed sweet potato, carrot, or pumpkin), 221 (46.4%) (Figure 2).

### Anemia and associated factors during pregnancy

Nearly two out of ten participants 103 (21.6%, 95%CI 18.2–25.6%) were diagnosed as mildly, moderately, or severely anemic. Of the anemic, 71 (68.9%) of respondents were mildly anemic and 32 (31.1%) were moderately anemic. Respondents who received IFA supplements in the current pregnancy were 66% less likely to be anemic than those who did not receive IFA, AOR (95% CI), 0.34 (0.19, 0.61). Women in the age group 20–29 years were 59% less likely to be anemic during pregnancy than older ages (≥ 30 years), AOR (95% CI), 0.41 (0.24, 0.69). Respondents who were in low educational attainment (elementary level) were 1.8 times more likely to be anemic in pregnancy than those who had attained college or above education, AOR (95% CI), 1.84 (1.03, 3.31).

Women with a history of malaria infection in the current pregnancy were 4 times more likely to be anemic than those who had no malaria infection, AOR (95% CI), 4.13 (1.95, 8.75). Women who slept under an insecticide-treated net the last night before the day of the survey were 53% less likely to be anemic in the current pregnancy than their counterparts, AOR (95% CI), 0.47 (0.29, 0.80) (Table 3).

**Table 3:** Adjusted and unadjusted logistic regression model that shows statistical associations between anemia status and characteristics among pregnant women, Arba Minch district, South Ethiopia, *n* = 476, January 2025.

Variable		Anemia status		COR (95%CI)	AOR (95%CI)	*P* value
		Yes, *n* (%)	No, *n* (%)			
IFA received in the current pregnancy	Yes	29 (12.6)	202 (87.4)	0.33 (0.21, 0.53)*	0.34 (0.19, 0.61)**	<0.001
	No	74 (30.2)	171 (69.8)	1	1	
Age of women (years)	15–19	8 (23.5)	26 (76.5)	0.75 (0.31, 1.78)	0.66 (0.25, 1.74)	0.40
	20–29	53 (17.8)	245 (82.2)	0.52 (0.33, 0.84)*	0.41 (0.24, 0.69)**	0.001
	>29	42 (29.2)	102 (70.8)	1	1	
MDD score	Not adequate	33 (17.9)	151 (82.1)	0.69 (0.44, 1.1)*	0.83 (0.49, 1.40)	0.49
	Adequate	70 (24.0)	222 (76.0)	1	1	
Women Education status	Elementary	52 (29.5)	124 (70.5)	1.88 (1.11, 3.19)*	1.84 (1.03, 3.31)**	0.041
	High School	24 (15.8)	128 (84.2)	0.84 (0.46, 1.54)	0.96 (0.50, 1.86)	0.91
	College	27 (18.2)	121 (81.8)	1	1	
History of malaria infection in the current pregnancy	Yes	18 (29.0)	44 (71.0)	1.58 (0.87, 2.88)*	4.13 (1.95, 8.75)**	<0.001
	No	85 (20.5)	329 (79.5)	1	1	
Health Insurance	Insured	26 (15.2)	145 (84.8)	0.53 (0.32, 0.87)*	0.60 (0.32, 1.12)	0.11
	Not insured	77 (25.2)	228 (74.8)	1	1	
Slept last night under ITN	Yes	60 (17.3)	286 (82.7)	0.42 (0.27, 0.67)*	0.47 (0.29, 0.80)**	0.005
	No	43 (33.1)	87 (66.9)	1	1	
Meal frequency	2 or 3 meals	53 (27.6)	139 (72.4)	2.74 (1.32, 5.71)*	2.17 (0.98, 4.82)	0.05
	3 meals, 1 snack	40 (19.8)	162 (80.2)	1.78 (0.84, 3.75)*	1.56 (0.71, 3.42)	0.27
	3 meals, 2+ snack	10 (12.2)	72 (87.8)	1	1	

* Significant by unadjusted logistic regression (*p* < 0.25).

** Atatistically significant associations (*p* < 0.05) or (*p* < 0.001).

IFA, iron folic acid; ITN, insecticide treated net; $US, united states dollar; BMI, body mass index.

## Discussions

This study observed various factors which had association with anemia during pregnancy. The respondent's IFA intake in the current pregnancy, age, education level of women, history of malaria infection in the current pregnancy, and nighttime sleeping under ITN were statistical predictors of anemia in pregnancy. However, variables such as women's MDD score, membership in community-based health insurance, and meal frequency in pregnancy were statistically significantly associated with the anemia during pregnancy in the unadjusted logistic regression model only.

This study revealed that IFA intake during antenatal visit were protective against anemia in pregnancy. This was in line with the evidence from the study in Ethiopia (19, 32) and elsewhere (15). This statistical association could possibly show protection of maternal dietary iron and folate deficiency through supplementation helped in maternal anemia prevention. Most women in this study had received plant source foods and this might have influenced on anemia status. This was because plant source foods mainly have non-heme iron which has low bioavailability and contains antinutrient inhibitors (such as, iron) of phytates (10, 33, 34), tannins, oxalates, saponins, and others (34). Even-though caffeine (which is an active component in the tea, cocoa, and coffee) is a phytoceuticals (antioxidant, anti-free radicals, or anti-inflammatory agent) (35, 36), it could affect the bioavailability of iron as an adverse effect (34, 37, 38). This was because more than half of the respondents (56%) reported as received tea or coffee within 30 min after consuming foods. The finding was in-line with the fact that IFA supplementation reduces anemia risk as reported by the World Bank group investment framework for meeting the global nutrition target to achieve 2025 mission of 50% anemia reduction in reproductive age women (1). More importantly, a recent Ethiopian nationwide survey also observed improvement in IFA intake over time and a reduction in anemia incidences in pregnant women of Ethiopia (6).

In this study, evidence was limited to declare statistical association of W-MDD score and anemia in pregnancy. The finding was not consistent with reports elsewhere (18, 19) in that statistical association was observed between anemia and inadequate dietary diversity score. The micronutrients (such as, iron, folate, and others) bioavailability inhibitors in plant foods (10, 33, 34) and consumption of tea (34, 37, 38) immediately after food consumption are another important factor that could have affected the influence of dietary diversity on the outcome variable. This was because majority of participants consumed plant source foods. Moreover, avoiding some foods in pregnancy due to reasons (Table 2) could have played a role to affect the effect of dietary diversity on the outcome.

In the current study, increasing maternal age was statistically associated with anemia. This was consistent with a recent meta-analysis estimate in Egypt (15). However, evidence was limited according to a pocket study reported from Oromia region of Ethiopia (39). This positive relationship could probably show that being an old age pregnancy (age ≥30 years) could be one of the risk pregnancies for anemia among others.

As revealed in the current study, higher educational attainment of women was protective against anemia in pregnancy. A recent Ethiopian Demography and Health survey report of 2016 (27) and a cohort study among pregnant women from 8 provinces of China showed similar results as anemia prevalence was decreasing with increasing education level of women (40). When women become highly educated, they will be healthier (41). This possibly shows that better health-related awareness for well-being and health-related self-protection practices among educated women may have influenced their choice for anemia risk protective practices in pregnancy, as justified elsewhere (41, 42).

Pregnant women who had a history of malaria infection any time in the current pregnancy were at risk for anemia in pregnancy. Similarly, in the place where there was high malaria risk, the risk for anemia was a high in Nigeria (43). Other evidence in Kenyan coast and Tanzania supported the relationship too (44, 45). This was due to excess removal of maternal erythrocytes by Plasmodium species (46) in that maternal hemoglobin concentration will be reduced.

The insecticide treated bed net (ITN) is a mosquito repellent and/or killer bed net made of mainly polyester or polyethylene (47). The combined net was more effective than uncombined (47). Women who slept last night under ITN were protected against anemia in pregnancy. This was possibly related to a lower risk of malaria infection for women who slept under ITN even though the study locality was known for high malaria prevalence in Ethiopia. The CDC and the Malaria Consortium stated that using ITN was the most effective and most used malaria preventive strategy in the areas where malaria was prevalent and endemic, being a barrier to mosquito bites and having an insecticidal effect (48, 49). Women who were infected with malaria in pregnancy were more likely to be anemic in pregnancy, as suggested elsewhere (43–45, 50).

## Strengths and limitations

### Strengths

Using the health facility laboratory result of blood hemoglobin level to access participant anemia data was useful to minimize recall bias. Mixed use of behavioral factors (ITN, malaria status, diet, and others) and clinical factors (such as IFA) to show association with anemia status is comprehensive.

### Limitations

None use of serum ferritin, serum iron, soluble transferrin receptors biomarkers, or red cell morphology (for vitamin A, B9, B6, B12, or riboflavin deficiency) due to resource limitations might have affected the result to differentiate iron or vitamin deficiency anemia (51). The participant selection, social desirability and recall bias could have affected the result. The selection bias was minimized using random selection with proportional allocation of respondents. The social desirability bias was minimized using objective clarity to the respondents before the start of interview. The recall bias was minimized by limited recall to the last 24-hours of the food consumed by the respondent. The results of this study could have been affected by the exclusion of non-antenatal visiting pregnant women who were at home during the study period. Design related causal association was another limitation.

## Conclusions

The substantial number of respondents were anemic in the study region. Supplementation with iron and folic acid during pregnancy lowers the occurrence of anemia in pregnancy. Women during antenatal follow-up should be encouraged to take IFA supplements. Moreover, sleeping under ITN and other malaria prevention interventions should be advised to pregnant women to be protected from anemia in pregnancy.

## Data Availability

The raw data supporting the conclusions of this article will be made available by the authors, without undue reservation.

## Footnotes

^1^South Ethiopia Region Health Bureau administration office. South Ethiopia Region Administrative Profile. September 2023 (unpublished).

## References

[1] World Bank AA Walters D Kakietek J Eberwein JD Shekar M. An Investment Framework for Meeting the Global Nutrition Target for Anemia (2017).

[2] The World Bank. Prevalence of anemia among pregnant women in the world, 2000–2009. Available at: https://data.worldbank.org/indicator/SH.PRG.ANEM (Accessed April 30, 2024).

[3] Karami M Chaleshgar M Salari N Akbari H Mohammadi M. Global prevalence of anemia in pregnant women: a comprehensive systematic review and meta-analysis. Matern Child Health J. (2022) 26(7):1473–87. 10.1007/s10995-022-03450-1 35608810

[4] WHO/UNICEF. The Extension of the 2025 Maternal, Infant and Young Child Nutrition Targets to 2030 WHO/UNICEF. (2014). Discussion paper file:///C:/Users/user/Dropbox/PC/Downloads/UNICEF-WHO-discussion-paper-extension-targets-2030.pdf.

[5] Kassa GM Muche AA Berhe AK Fekadu GA. Prevalence and determinants of anemia among pregnant women in Ethiopia; a systematic review and meta-analysis. BMC Hematol. (2017) 17(1):17. 10.1186/s12878-017-0090-z 29075500 PMC5646153

[6] EPHI. Ethiopian National Food and Nutrition Strategy Baseline Survey. (2023). p. 1–30. Available at: https://ephi.gov.et/food-and-nutrition-strategy-baseline-survey-preliminary-finding-dissemination-workshop/ (Accessed June 17, 2024).

[7] World Bank GROUP. Prevalence of anemia among women of reproductive age (% of women ages 15–49). (2020). Available at: https://genderdata.worldbank.org/en/indicator/sh-anm-allw-zs (Accessed December 20, 2024).

[8] FAO. Minimum Dietary Diversity for Women. Rome: FAO Knowledge Repository. (2021). 10.4060/cb3434en

[9] Simone M. Gie, Giles Hanley-Cook, Sara Hoogerwerf, Juan Pablo Parraguez, Bridget Holmes. Food and Agriculture Organization of the United Nations. Rome: Minimum Dietary Diversity for Women (MDD-W) (2024).

[10] Piskin E Cianciosi D Gulec S Tomas M Capanoglu E. Iron absorption: factors, limitations, and improvement methods. ACS Omega. (2022) 7(24):20441–56. 10.1021/acsomega.2c01833 35755397 PMC9219084

[11] Czerwonka M Tokarz A. Iron in red meat-friend or foe. Meat Sci. (2017) 123:157–65. 10.1016/j.meatsci.2016.09.012 27744145

[12] Monsen ER. Iron nutrition and absorption: dietary factors which impact iron bioavailability1. J Am Diet Assoc. (1988) 88(7):786–90. 10.1016/S0002-8223(21)07902-5 3290310

[13] Ems T Lucia KS Huecker MR. Biochemistry, iron absorption. In: StatPearls [Internet]. Treasure Island, FL: StatPearls Publishing (2025). 28846259

[14] Peter M Amrit Roshin JE Varghese J Venkatesan P. Dietary factors that influence iron deficiency anemia in India. Indian J Public Health. (2025) 69(1):100–3. 10.4103/ijph.ijph_1441_23 40214326

[15] Azzam A Khaled H Alrefaey AK Basil A Ibrahim S Elsayed MS Anemia in pregnancy: a systematic review and meta-analysis of prevalence, determinants, and health impacts in Egypt. BMC Pregnancy Childbirth. (2025) 25(1):29. 10.1186/s12884-024-07111-9 39810098 PMC11731563

[16] Shitie D Zewde T Molla Y. Anemia and other hematological profiles of pregnant women attending antenatal care in Debre Berhan Referral Hospital, North Shoa, Ethiopia. BMC Res Notes. (2018) 11(1):704. 10.1186/s13104-018-3805-8 30290844 PMC6173918

[17] Gudeta TA Regassa TM Belay AS. Magnitude and factors associated with anemia among pregnant women attending antenatal care in Bench Maji, Keffa and Sheka zones of public hospitals, southwest, Ethiopia, 2018: a cross -sectional study. PLoS One. (2019) 14(11):e0225148. 10.1371/journal.pone.0225148 31751368 PMC6872185

[18] Gibore NS Ngowi AF Munyogwa MJ Ali MM. Dietary habits associated with anemia in pregnant women attending antenatal care services. Curr Dev Nutr. (2021) 5(1):nzaa178. 10.1093/cdn/nzaa178 33501404 PMC7809361

[19] Lebso M Anato A Loha E. Prevalence of anemia and associated factors among pregnant women in southern Ethiopia: a community based cross-sectional study. PLoS One. (2017) 12(12):e0188783. 10.1371/journal.pone.0188783 29228009 PMC5724831

[20] Ba DM Ssentongo P Liao D Du P Kjerulff KH. Non-iodized salt consumption among women of reproductive age in sub-saharan Africa: a population-based study. Public Health Nutr. (2020) 23(15):2759–69. 10.1017/S1368980019003616 31915084 PMC10200610

[21] UNOCHA. Humanitarian data exchange 2021. Available at: https://data.humdata.org/dataset/?groups=eth&res_format=GeoTIFF&res_format=Geodatabase&q=&sort=last_modified%20desc&ext_page_size=25 (Accessed June 21, 2024).

[22] Lim SS Allen K Bhutta ZA Dandona L Forouzanfar MH Fullman N Measuring the health-related sustainable development goals in 188 countries: a baseline analysis from the global burden of disease study 2015. Lancet. (2016) 388(10053):1813–50. 10.1016/S0140-6736(16)31467-2 27665228 PMC5055583

[23] Ethiopian Statistics Service. The population development in Southern Region Ethiopia, the 2007 census atlas and current official projections at July, 2022. Available at: https://www.citypopulation.de/en/ethiopia/admin/ET07__southern/ (Accessed June 22, 2024).

[24] Argaw D Hussen Kabthymer R Birhane M. Magnitude of anemia and its associated factors among pregnant women attending antenatal care in Southern Ethiopia: a cross-sectional study. J Blood Med. (2020) 11:335–44. 10.2147/JBM.S264369 33117019 PMC7553252

[25] Henderson RH Sundaresan T. Cluster sampling to assess immunization coverage: a review of experience with a simplified sampling method. Bull World Health Organ. (1982) 60(2):253–60. https://pmc.ncbi.nlm.nih.gov/articles/PMC2535957/6980735 PMC2535957

[26] Science direct. The design effect. (2025). Available at: https://www.sciencedirect.com/topics/mathematics/design-effect. (Accessed January 03, 2025).

[27] Central Statistical Agency CSAE, Icf. Ethiopia Demographic and Health Survey 2016. Addis Ababa, Ethiopia: CSA and ICF (2017).

[28] Gebreweld A Tsegaye A. Prevalence and factors associated with anemia among pregnant women attending antenatal clinic at St. Paul's Hospital millennium medical college, Addis Ababa, Ethiopia. Adv Hematol. (2018) 2018(1):3942301. 10.1155/2018/3942301 30245724 PMC6136568

[29] Bitew ZW Alemu A Ayele EG Worku T. Dietary diversity and practice of pregnant and lactating women in Ethiopia: a systematic review and meta-analysis. Food Sci Nutr. (2021) 9(5):2686–702. 10.1002/fsn3.2228 34026082 PMC8116864

[30] UNHCR. The United Nations Refugee agency, MODULE 3: ANAEMIA, adjustment for altitude. (2012). Available at: https://www.unhcr.org/sens/wp-content/uploads/sites/155/2020/09/Tool_05_SENS_ANAEMIA_Hb_Adjustment_for_Altitude_v3.pdf (Accessed June 21, 2024).

[31] World Health Organization. Disease outbreak news: geographical distribution of Malaria cases as of 20 October, 2024. Malaria in Ethiopia. (2024). Available at: http://www.who.int/emergencies/disease-outbreak-news/item/2024-DON542 (Accessed December 23, 2024).

[32] Kuma MN Tamiru D Belachew T. Hemoglobin level and associated factors among pregnant women in rural southwest Ethiopia. Biomed Res Int. (2021) 2021:9922370. 10.1155/2021/9922370 34104652 PMC8159627

[33] Beck KL Conlon CA Kruger R Coad J. Dietary determinants of and possible solutions to iron deficiency for young women living in industrialized countries: a review. Nutrients. (2014) 6(9):3747–76. 10.3390/nu6093747 25244367 PMC4179187

[34] Shahid R Iahtisham Ul H Mahnoor Awan KA Iqbal MJ Munir H Diet and lifestyle modifications for effective management of polycystic ovarian syndrome (PCOS). J Food Biochem. (2022) 46(7):e14117. 10.1111/jfbc.14117 35199348

[35] Awan KA Iahtisham Ul H Butt MS Munir H Sultan W Yaqoob S Restorative potential of Phoenix dactylifera fruit and extract against oxidative stress mediated cardiac dysfunction in rodent experimental modeling. J Funct Foods. (2025) 128:106845. 10.1016/j.jff.2025.106845

[36] Sreenathkumar S. Current updates on global phytoceuticals and novel phyto drug delivery system in herbal medicine. In: El-Shemy H, editor. Natural Drugs from Plants. Rijeka: IntechOpen (2021). p. 320–31.

[37] Ahmad Fuzi SF Koller D Bruggraber S Pereira DI Dainty JR Mushtaq S. A 1-h time interval between a meal containing iron and consumption of tea attenuates the inhibitory effects on iron absorption: a controlled trial in a cohort of healthy UK women using a stable iron isotope. Am J Clin Nutr. (2017) 106(6):1413–21. 10.3945/ajcn.117.161364 29046302

[38] Sung ES Choi CK Kim NR Kim SA Shin MH. Association of coffee and tea with ferritin: data from the Korean national health and nutrition examination survey (IV and V). Chonnam Med J. (2018) 54(3):178–83. 10.4068/cmj.2018.54.3.178 30288374 PMC6165914

[39] Zillmer K Pokharel A Spielman K Kershaw M Ayele K Kidane Y Predictors of anemia in pregnant women residing in rural areas of the oromiya region of Ethiopia. BMC Nutr. (2017) 3(1):65. 10.1186/s40795-017-0166-y 32153845 PMC7050732

[40] Qiao Y Di J Yin L Huang A Wei Z Hu H Prevalence and influencing factors of anemia among pregnant women across first, second and third trimesters of pregnancy in monitoring areas, from 2016 to 2020: a population-based multi-center cohort study. BMC Public Health. (2024) 24:1–9. 10.1186/s12889-024-8610-x 38649895 PMC11034068

[41] UN Department of Economics and Social Affairs. Better education brings longer, healthier lives to women. (2015). Available at: https://www.un.org/development/desa/en/news/statistics/gender-stat.html (Accessed December 13, 2024).

[42] Institute of Medicine Committee on Health L. In: Nielsen-Bohlman L Panzer AM Kindig DA, editors. Health Literacy: A Prescription to End Confusion. Washington, DC: National Academies Press (US) (2004). p. 1–367. 25009856

[43] Ugwu EO Dim CC Uzochukwu BS Iloghalu EI Ugwu AO. Malaria and anaemia in pregnancy: a cross-sectional study of pregnant women in rural communities of Southeastern Nigeria. Int Health. (2014) 6(2):130–7. 10.1093/inthealth/ihu009 24664630

[44] Shulman CE Dorman EK Bulmer JN. Malaria as a cause of severe anaemia in pregnancy. Lancet. (2002) 360(9331):494. 10.1016/S0140-6736(02)09662-9 12241758

[45] Msuya SE Hussein TH Uriyo J Sam NE Stray-Pedersen B. Anaemia among pregnant women in northern Tanzania: prevalence, risk factors and effect on perinatal outcomes. Tanzan J Health Res. (2011) 13(1):33–9. 10.4314/thrb.v13i1.60881 24409645

[46] Ekvall H. Malaria and anemia. Curr Opin Hematol. (2003) 10(2):108–14. 10.1097/00062752-200303000-00002 12579035

[47] CDC. Malaria. Insecticide-Treated Nets April 2, 2024.

[48] Malaria Consortium. Advocacy Brief 2016. Malaria prevention through insecticide treated nets.

[49] CDC. Malaria. (2024).

[50] WHO. Nutrition and nutrition-related health and development data, anemia. Available at: https://www.who.int/data/nutrition/nlis/info/anaemia. (Accessed October 10, 2024).

[51] Northrop-Clewes CA Thurnham DI. Biomarkers for the differentiation of anemia and their clinical usefulness. J Blood Med. (2013) 4:11–22. 10.2147/JBM.S29212 23687454 PMC3610441

[52] FDRE Ministry of Science and Technology. National Research Ethics Review Guideline Fifth Edition. (2015). Available at: https://www.studocu.com/row/u/12433566?sid=01714558687 (Accessed December 13, 2024).

